# 1950. Immune-sensitization to *Mycobacterium tuberculosis* Among Young Ugandan Children With and Without Tuberculosis

**DOI:** 10.1093/ofid/ofad500.104

**Published:** 2023-11-27

**Authors:** Jesus Gutierrez, LaShaunda Malone, John Mukisa, Ezekiel Mupere, Catherine Stein, Christina Lancioni

**Affiliations:** Case Western Reserve University, Cleveland, OH; Case Western Reserve University, Cleveland, OH; Makerere University, Kampala, Kampala, Uganda; Makerere University, Kampala, Kampala, Uganda; Case Western Reserve University, Cleveland, OH; Oregon Health & Science University, Portland, Oregon

## Abstract

**Background:**

*Mycobacterium tuberculosis* (*Mtb*) remains a leading cause of pediatric morbidity and mortality worldwide. As TB develops quickly following exposure in young children, identifying clinical, diagnostic, and epidemiologic factors associated with progression to pediatric TB is an important step towards early diagnosis, and prioritization for preventive therapy. Our objectives are to compare epidemiologic risk scores (ERS), and tests of immune-sensitization to *Mtb*, among young children with known *Mtb* exposure who did and did not develop TB.

**Methods:**

Using a household contact (HHC) study in Kampala, Uganda, we recruited 75 children ≤ 5 years living with an adult with recently confirmed TB, performed a complete TB diagnostic evaluation, and assigned children into two baseline cohorts: asymptomatic with negative diagnostic studies (PedAS) versus symptomatic (PedTB). All children underwent tuberculin skin test (TST) and interferon gamma release assay (IGRA) testing at enrollment, and detailed epidemiologic and clinical characteristics were used to compute ERS for *Mtb* exposure (Figure 1). Comparisons were performed using chi-square test, T-test, Fisher’s exact test and Mann–Whitney *U* test.

**Figure 1.**
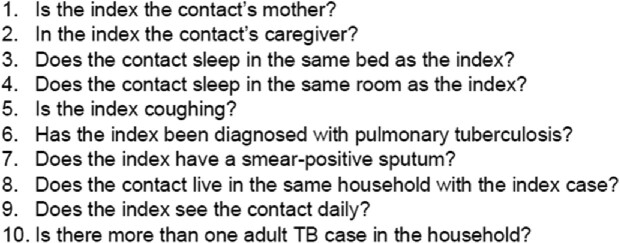
Epidemiologic Risk Score (ERS)

The ERS includes 10 indicators of risk of Mtb infection. The score ranges from 0 to 10, and it is composed as a sum across all the above variables with 1 equaling the presence of each particular risk factor. A higher score indicates a higher risk.

**Results:**

Of 75 children, 45 (60%) were classified as PedAS and 30 (40%) as PedTB. The two groups did not differ in terms of age, sex, ERS, or presence of BCG scar (Table 1). IGRA positivity also did not differ between groups (27.3% vs. 50%, p-value = 0.09), and when stratified by age, only 30% of PedTB participants < 2 years had a positive IGRA (Table 2). There was no difference in ERS by IGRA status. Indeterminate IGRA results occurred in 3/75 participants. Conversely, TST was more likely to be positive categorically (40% vs. 70%, p-value = 0.02) and quantitatively (0mm vs. 11.9mm, p-value = 0.02) among those in the PedsTB cohort. There was no difference in ERS by TST status.

**Table 1.**
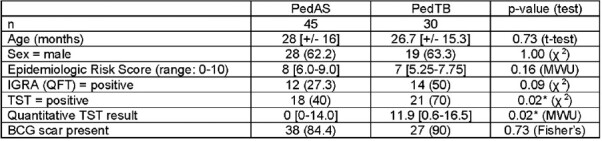
Comparing baseline cohorts: Asymptomatic for TB (PedAS) vs. Symptomatic for TB (PedTB)

Assignment of baseline cohorts was based on presence of signs and symptoms of TB disease, chest X-ray findings, and microbiologic testing (AFB smear and culture; GeneXpert Ultra) of two induced sputum samples. Counts (percentages), means [+/- standard deviation], or median [quartiles]. χ 2: Chi-squared test. MWU: Mann–Whitney U test. QFT: QuantiFERON-TB Gold. * Statistically significant at p<0.05.

**Table 2.**
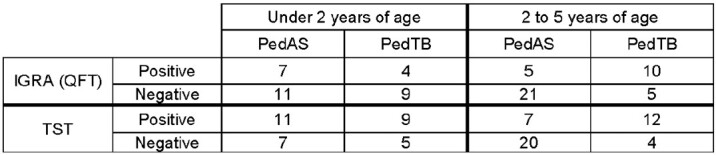
Comparing immune-sensitization of Asymptomatic for TB (PedAS) and Symptomatic for TB (PedTB) cohorts stratified by age

Assignment of baseline cohorts was based on presence of signs and symptoms of TB disease, CXR findings, and microbiologic testing (AFB smear and culture; GeneXpert Ultra) of two induced sputum samples. QFT: QuantiFERON-TB Gold.

**Conclusion:**

In this cohort of young, Ugandan children with known *Mtb*-exposure, commonly used epidemiologic and clinical characteristics quantified as ERS, and IGRA testing, were not useful in distinguishing between children with TB diseases versus asymptomatic exposure. However, TST was significantly different between cohorts, suggesting that TST is valuable for detection of immune-sensitization to *Mtb* in young children who develop TB.

**Disclosures:**

**All Authors**: No reported disclosures

